# Purification and Characterization of a Novel NAD(P)^+^-Farnesol Dehydrogenase from *Polygonum minus* Leaves

**DOI:** 10.1371/journal.pone.0143310

**Published:** 2015-11-23

**Authors:** Nor-Ain-Shahajar Ahmad-Sohdi, Ahmad-Faris Seman-Kamarulzaman, Zeti-Azura Mohamed-Hussein, Maizom Hassan

**Affiliations:** 1 Institute of Systems Biology, Universiti Kebangsaan Malaysia (UKM), 43600 UKM, Bangi, Selangor, Malaysia; 2 School of Biosciences and Biotechnology, Faculty of Science and Technology, Universiti Kebangsaan Malaysia, 43600 UKM, Bangi, Selangor, Malaysia; National Research Council of Italy, ITALY

## Abstract

Juvenile hormones have attracted attention as safe and selective targets for the design and development of environmentally friendly and biorational insecticides. In the juvenile hormone III biosynthetic pathway, the enzyme farnesol dehydrogenase catalyzes the oxidation of farnesol to farnesal. In this study, farnesol dehydrogenase was extracted from *Polygonum minus* leaves and purified 204-fold to apparent homogeneity by ion-exchange chromatography using DEAE-Toyopearl, SP-Toyopearl, and Super-Q Toyopearl, followed by three successive purifications by gel filtration chromatography on a TSK-gel GS3000SW. The enzyme is a heterodimer comprised of subunits with molecular masses of 65 kDa and 70 kDa. The optimum temperature and pH were 35°C and pH 9.5, respectively. Activity was inhibited by sulfhydryl reagents, metal-chelating agents and heavy metal ions. The enzyme utilized both NAD^+^ and NADP^+^ as coenzymes with *K*_m_ values of 0.74 mM and 40 mM, respectively. *Trans*, *trans*-farnesol was the preferred substrate for the *P*. *minus* farnesol dehydrogenase. Geometrical isomers of *trans*, *trans*-farnesol, *cis*, *trans*-farnesol and *cis*, *cis*-farnesol were also oxidized by the enzyme with lower activity. The *K*_m_ values for t*rans*, *trans*-farnesol, *cis*, *trans*-farnesol and *cis*, *cis*-farnesol appeared to be 0.17 mM, 0.33 mM and 0.42 mM, respectively. The amino acid sequences of 4 tryptic peptides of the enzyme were analyzed by MALDI-TOF/TOF-MS spectrometry, and showed no significant similarity to those of previously reported farnesol dehydrogenases. These results suggest that the purified enzyme is a novel NAD(P)^+^-dependent farnesol dehydrogenase. The purification and characterization established in the current study will serve as a basis to provide new information for recombinant production of the enzyme. Therefore, recombinant farnesol dehydrogenase may provide a useful molecular tool in manipulating juvenile hormone biosynthesis to generate transgenic plants for pest control.

## Introduction

Juvenile hormones (JHs) are a family of sesquiterpenes that play important roles in the development, metamorphosis, reproduction, polyphenism, and behavioral changes of insects [1]. Due to these properties, JHs have attracted attention as safe and selective targets for the design and development of environmentally friendly and biorational insecticides [2]. Several JH analogs have been reported, such as ethyl 4-[2-(tert-butylcarbonyloxy)butoxy]benzoate, fluoromevalonate, ethyl (E)-3-methyl-2-dodecenoate, fluvastatin and methoprene though none of these compounds has proven to be sufficiently active for practical use in pest control [3–6].

The biosynthetic pathway of juvenile hormone III (JH III, methyl-10*R*,11-epoxy-3,7,11-trimethyl-2*E*, 6*E*-dodecadienoate) is well conserved in insects [7]. The early steps in the biosynthetic pathway of insect JH III include the mevalonate pathway from acetyl-CoA to farnesyl pyrophosphate, a conserved pathway in both vertebrates and invertebrates [8]. Farnesyl pyrophosphate synthase catalyzes the synthesis of farnesyl pyrophosphate from dimethylallyl diphosphate and isopentenyl pyrophosphate [9]. Farnesol is synthesized by farnesyl pyrophosphatase, [10] and then oxidized by farnesol dehydrogenase to produce farnesal [11, 12]. Subsequently, farnesal is oxidized most probably by farnesal dehydrogenase to form farnesoic acid [13]. The last two steps of the JH III biosynthetic pathway depend on the particular order of insect. In Lepidoptera, a P450 monooxygenase converts farnesoic acid to JH acid which is subsequently methylated by JH acid O-methyltransferase to form JH III [14]. However, in orthopteran and dictyopteran insects, the methylation reaction is preceded by an epoxidation reaction [8]. In comparison to the number of enzymatically well characterized enzymes from insect JH III biosynthetic pathway [12, 15–18], enzymes involve in plant JH III biosynthetic pathway are not well elucidated. JH III and its biosynthetic precursor in insects, methyl farnesoate, were also identified in the sedges, *Cyperus iria* L. and *C*. *aromaticus* [19]. Inhibitor and precursor feeding studies suggest that the later steps of the JH III biosynthesis in *C*. *iria* are similar to those in the insect pathway [20]. In addition, enzyme activities of farnesyl pyrophosphate synthase, farnesyl pyrophatase, farnesol dehydrogenase and methyltransferase which involved in JH III biosynthesis were detected in several plants [11, 21–27]. High concentration of JH III in *C*. *iria* roots and its present throughout development indicated that this compound plays important roles in plant mechanism through plant-insect, plant-plant, or other interaction [13]. Furthermore, the crude extract of *C*. *aromaticus* cultured cells which contained JH III showed growth inhibitory against *Aedes aegypti and A*. *albopictus*. The economic importance of insect hormones in plants was highlighted by their ability to inhibit the development and reproduction of insect herbivores [28]. Therefore, more detailed understanding of the enzymes in JH III biosynthetic and metabolic pathways in plant will be useful for the development of new approaches towards integrated pest management using recombinant DNA technology [29] by deployment of the genetically transformed plants for pest control [30].

To elucidate the JH III biosynthetic pathway in plant, we investigated enzymes participating in this sesquiterpene metabolism pathway in *Polygonum minus*. This species belongs to the Polygonaceae family and possesses a wide range of medicinal properties [31–35]. Farnesol and farnesal have been identified in the essential oils of *Polygonum* sp. [36, 37]. Moreover, enzyme activities of farnesyl pyrophosphate synthase, farnesol dehydrogenase and farnesal dehydrogenase have also been detected in cell-free extracts of *P*. *minus*. Farnesol dehydrogenase is an enzyme that catalyzes the oxidation of farnesol to farnesal [38]. Farnesol dehydrogenase activity has been reported to be present in *Arabidopsis thaliana* [26], *Ipomoea batatas* [11], and in the corpora allata glands of *Aedes aegypti* [12]. However, only farnesol dehydrogenase from *A*. *aegypti* has been purified to homogeneity. In addition, the existing papers lack of the information on the enzyme recognition of substrate specificity. Based on the current state of research, the nature of substrates that are specifically oxidized by farnesol dehydrogenase has remained poorly understood. This paper reports the purification and characterization of farnesol dehydrogenase enzyme from *P*. *minus* leaves. Purification was achieved using ion exchange and gel filtration chromatographies. To the best of our knowledge, this is the first report that demonstrates the utilization of both NAD^+^ and NADP^+^ as coenzymes by a farnesol dehydrogenase enzyme. The deployment of transgenic plant with farnesol dehydrogenase enzyme will be beneficial for use in manipulating juvenile hormone biosynthesis in plants. Thus, offers an alternative method for controlling population of insect pest by means of non-toxic, selectively acting and biorationally safe practice [28].

## Materials and Methods

### Plant materials and chemicals

The *P*. *minus* leaves were obtained from plants growing in an experimental plot at the Institute of Systems Biology of Universiti Kebangsaan Malaysia (UKM). *Trans*, *trans*-farnesol (*trans*,*trans*-3,7,11-trimethyl-2,6,10-dodecatrien-1-ol) was obtained from Alfa Aesar (Ward Hill, MA). *Cis*, *trans*-farnesol was purchased from Tokyo Chemical Industry (TCI) (Tokyo, Japan). *Cis*, *cis*-farnesol was obtained from Echelon Bioscience, Inc. (Salt Lake City, UT). DEAE-Toyopearl 650M, SuperQ Toyopearl 650M, SP Toyopearl 650M and TSK-gel GS3000SW were purchased from Tosoh (Tokyo, Japan), whilst standard proteins for gel filtration were obtained from Bio-Rad (Hercules, CA). All other reagents were analytical-grade commercial products. Water-insoluble chemicals were dissolved in absolute dimethyl sulfoxide or acetone, and subsequent dilutions were conducted in water. The presence of dimethyl sulfoxide or acetone in the reaction mixture had no effect on the enzyme activity.

### Extraction of farnesol dehydrogenase

Preparation of the cell-free extract was performed according to the method described by Hassan et al. [39] with slight modifications. Approximately 200 g (fresh weight) of *P*. *minus* leaves were frozen in liquid nitrogen and ground to a fine powder with a Waring blender. The frozen powder was immediately slurried with cold extraction buffer (100 mM tricine-NaOH buffer (pH 7.5) containing 2.5 mM of 2-mercaptoethanol (2-ME), 15% (v/v) of glycerol, 5 mM of thiourea, 1 mM of phenylmethylsulfonylfluoride (PMSF), 50% (w/w) Amberlite XAD-4 and 10% (w/v) polyvinylpolypyrrolidone (PVPP) for 15 min before being squeezed through four layers of cheesecloth. The homogenate was centrifuged at 20,000 × *g* at 4°C for 30 min to remove cell debris. The supernatant which was determined to contain farnesol dehydrogenase activity was used as the enzyme source.

### Protein measurement

Protein concentration was measured using the Lowry method [40] with bovine serum albumin as a standard. The proteins eluted from column chromatography were monitored by measuring absorbance at 280 nm.

### Enzyme assay

Farnesol dehydrogenase activity was measured by observing the increase in absorbance at 340 nm at 35°C. The standard reaction mixture (1.5 ml) contained 100 mM of glycine-NaOH buffer (pH 9.5), 1.0 mM of *trans*, *trans*-farnesol, 1.0 mM of NAD^+^ and an appropriate amount of enzyme. The reaction was initiated by addition of the enzyme. Enzyme activity was calculated using an extinction coefficient of 6,200 M^-1^ cm^-1^ for NADH. One unit of enzyme activity was defined as the amount of enzyme required to catalyze the formation of 1 μmol of NADH per min under the described assay conditions. Specific activity was defined as the units of enzyme activity per mg of protein.

### Purification of farnesol dehydrogenase

Purification of farnesol dehydrogenase was performed at 4°C. Throughout the purification procedure, 100 mM tricine-NaOH buffer (pH 7.5) containing 2.5 mM of 2-ME was used, unless otherwise stated. The flow rate during loading, washing, and elution was maintained at 1.5 ml/min unless stated. The cell-free extract (6600 mg protein) was put on a DEAE-Toyopearl 650M column (2.6 × 65 cm) equilibrated with the buffer. The column was washed with five column volumes of the buffer (15ml/fraction) and the unbound proteins with enzyme activity (300 ml) were collected. The fractions with enzyme activity from DEAE-Toyopearl were combined and applied to a SP-Toyopearl 650M column (1.6 × 37 cm) which had been equilibrated and washed with the buffer. Farnesol dehydrogenase activity was found in the unbound proteins. Fractions with farnesol dehydrogenase activity (15ml/fraction) from SP-Toyopearl were pooled (135 ml) and applied to a Super-Q Toyopearl 650M column (2.6 × 20 cm) that had been equilibrated with the buffer. The unbound proteins with enzyme activity were collected, concentrated with a Macrosep 10K Omega centrifugal devices (Pall Life Sciences, Ann Arbor, MI) and applied to a TSK-gel GS3000SW column (0.78 × 30 cm) equilibrated with the buffer. The protein was eluted with the buffer at a flow rate of 0.5 ml/min. Fractions with enzyme activity were pooled, concentrated using Macrosep 10K Omega centrifugal device and subjected to the same gel filtration procedure. Fractions showing farnesol dehydrogenase activity were pooled together, concentrated, and applied to a TSK-gel GS3000SW column for the third times. Finally, fractions containing enzyme activity were pooled and stored at -80°C until further use. The proteins eluted from column chromatography were monitored by measuring absorbance at 280 nm. The purity of the enzyme was determined by polyacrylamide gel electrophoresis (native-PAGE).

### Polyacrylamide gel electrophoresis

Native-PAGE electrophoresis was performed using a 12.5% polyacrylamide gel at pH 8.8, with the Laemmli buffer system without SDS [41]. The protein was silver-stained using the PlusOne silver staining kit (GE Healthcare, Uppsala, Sweden). Enzyme activity staining was performed according to the method described by Hassan et al. [39] with slight modifications. The activity staining of the gel was performed in the presence of 1.0 mM of *trans*, *trans*-farnesol in 100 mM of glycine-NaOH buffer (pH 9.5) containing 54 μM of 1-methoxy phenazine methosulphate, 0.3 mM of nitroblue tetrazolium, and 1.0 mM of NAD^+^ at 35°C for 2 h.

### Measurement of molecular mass and isoelectric point

The molecular mass of the enzyme was estimated by gel filtration on a TSK-gel GS3000SW column (0.75 × 30 cm) equilibrated with 0.1 M Tris-HCl buffer (pH 7.5) containing 2.5 mM of 2-ME. The purified enzyme (33 μg) was dialyzed against 3.0 L of 0.1 M Tris-HCl buffer (pH 7.5) containing 2.5 mM of 2-ME for 24 h and applied to the TSK-gel GS3000SW column. The proteins used for molecular-weight standards were thyroglobulin, γ-globulin, ovalbumin, myoglobin, and vitamin B_12_. SDS polyacrylamide gel electrophoresis (SDS-PAGE) was performed using a 12.5% polyacrylamide gel using the Laemmli method [41]. The PageRuler™ Prestained Protein Ladder, which has a standard-protein molecular-weight range of approximately 10–170 kDa (Product SM0671, Fermentas, St. Leon-Rot, Germany), was used as molecular marker. The protein was silver-stained as described previously.

Isoelectric focusing was performed with an 18 cm ReadyStrip IPG strip (pH 3–10) (GE Healthcare Bioscience, Uppsala, Sweden). The strip was passively rehydrated with 0.2 μg of purified farnesol dehydrogenase in rehydration buffer (8.0 M urea, 4% (w/v) CHAPS, 0.5% (v/v) of pH 3–10 ampholites, 30 mM 2-ME, and 0.002% bromophenol blue) for 12 h. The isoelectric focusing was carried out using Ethan IPGphor (GE Healthcare Bioscience, Uppsala, Sweden) according to the manufacturer’s instructions. The strip was silver stained.

### Protein identification by MALDI-TOF/TOF mass spectrometry (MALDI-TOF/TOF-MS)

Identification and analysis of the purified protein was carried out by peptide mass fingerprinting using MALDI-TOF/TOF mass spectrometry (MALDI-TOF/TOF-MS). The purified farnesol dehydrogenase was dialyzed against 100 mM Tris-HCl buffer (pH 7.5) overnight before the protein solution was sent to the Proteomics Facility, Medical Biotechnology Laboratory, Faculty of Medicine, University of Malaya, Kuala Lumpur, Malaysia for mass spectrometry analysis. In-solution trypsin digestion, protein extraction and mass spectrometry analysis by MALDI-TOF/TOF-MS were carried out according to protocols described by Sarah et al. [42] with slight modification. Protein was identified by the set of its proteolytic peptide masses using Peptide Mass Fingerprint option of Mascot software (Matrix Science, USA, http://www.matrixscience.com). Peptide mass profiles were searched and the peptides sequences were blasted against NCBI non-redundant (nr) database (http://blast.ncbi.nlm.nih.gov/Blast.cgi). The following combined parameters were used in NCBI searches: Viridiplantae was set as the organism, and the search was applied to other known full-length sequences of terpene alcohol dehydrogenases and benzyl alcohol dehydrogenases from *Persicaria minor* (nerol dehydrogenase; AFQ59973.1), *Castellaniella defragrans* (geraniol dehydrogenase; CCF55024.1), *Carpoglyphus lactis* (geraniol dehydrogenase; BAG32342.1), *Ocimum basilicum* (geraniol dehydrogenase; AAX83107.1), *A*. *thaliana* (Rossmann-fold NAD(P)-binding domain-containing protein; AEE86213.1), *A*. *aegypti* (NADP^+^-dependent farnesol dehydrogenase; ADB03640.1), *Lavandula x intermedia* (borneol dehydrogenase; AFV30207.1), *Picea abies* (cinnamyl alcohol dehydrogenase; CAA0597.1), *Fragaria x ananassa* (cinnamyl alcohol dehydrogenase; AAD10327.1), *Artemisia annua* (cinnamyl alcohol dehydrogenase; ACB54931.1), *Pseudomonas putida* (*p*-cumic alcohol dehydrogenase; AAB62297.1), *Mentha x piperita* ((-)-isopiperitenol dehydrogenase; AAU20370.1), *Streptomyces* sp. NL15-2K (coniferyl alcohol dehydrogenase; BAN09098.1), *A*. *thaliana* (allyl alcohol dehydrogenase; AAG50689.1), *Nicotiana tabacum* (allyl alcohol dehydrogenase; BAA89423.1), *P*. *putida* (aryl alcohol dehydrogenase; P39849.1), and *P*. *putida* (benzyl alcohol dehydrogenase; AAC32671.1).

### Effects of pH and temperature on farnesol dehydrogenase activity

The effect of pH on the activity of the purified farnesol dehydrogenase (0.2 μg) was studied with 1.0 mM of *trans*, *trans*-farnesol and 1.0 mM NAD^+^ as substrates at 35°C. Farnesol dehydrogenase activity was assayed at pH values ranging from 5.0 to 11.0 in 0.5 of increment. The following buffers were used at final concentration of 100 mM in the incubation mixture: citrate buffers (pH 5.0–6.0), potassium phosphate buffers (pH 6.5–7.5), glycine-NaOH buffers (pH 9.0–10.5), and carbonate buffers (pH 10.5–11.0). The enzyme activity was expressed as the percentage of the maximum activity. For the optimum temperature, the activity of farnesol dehydrogenase (0.2 μg) was measured under the standard assay conditions, except with the reaction temperature varied between 25–70°C. The enzyme activity was expressed as the percentage of the maximum activity. The temperature stability was determined by incubating the purified enzyme (0.2 μg) at temperature ranging from 25–70°C for 10 min at pH 7.5 (100 mM of tricine-NaOH buffer containing 2.5 mM of 2-ME). The residual farnesol dehydrogenase activity was assayed under the standard enzyme assay conditions. The enzyme activity was defined as the percentage of the maximum activity level.

### Effects of metal ions and inhibitors on the farnesol dehydrogenase activity

To determine the effects of inhibitors and metal ions on farnesol dehydrogenase activity, the purified enzyme (0.2 μg) was preincubated with various metal ions and inhibitors at a final concentration of 1.0 mM for 10 min at 35°C, followed by the standard enzyme assay as described before. The effect of inhibitors tested include 2,2-dipyridil, iodoacetamide, sodium azide, 5,5’-dithiobis (2-nitrobenzoic acid), *p*-chloromercuribenzoate, EDTA, 1, 10-phenanthroline and the metal ions tested include Li^+^, Ca^+^, Ag^+^, Zn^2+^, Cu^2+^, Mg^2+^ and Fe^3+^. These inhibitors and metal ions were widely used in many previous reports of alcohol dehydrogenase [11, 39, 43–45], and thus were selected for this study. The enzyme activity obtained from the reaction mixture without any extra ion or inhibitor was taken as a control, corresponding to 100% relative activity.

### Substrate specificity and Michaelis-Menten constants

**Fig 1:**
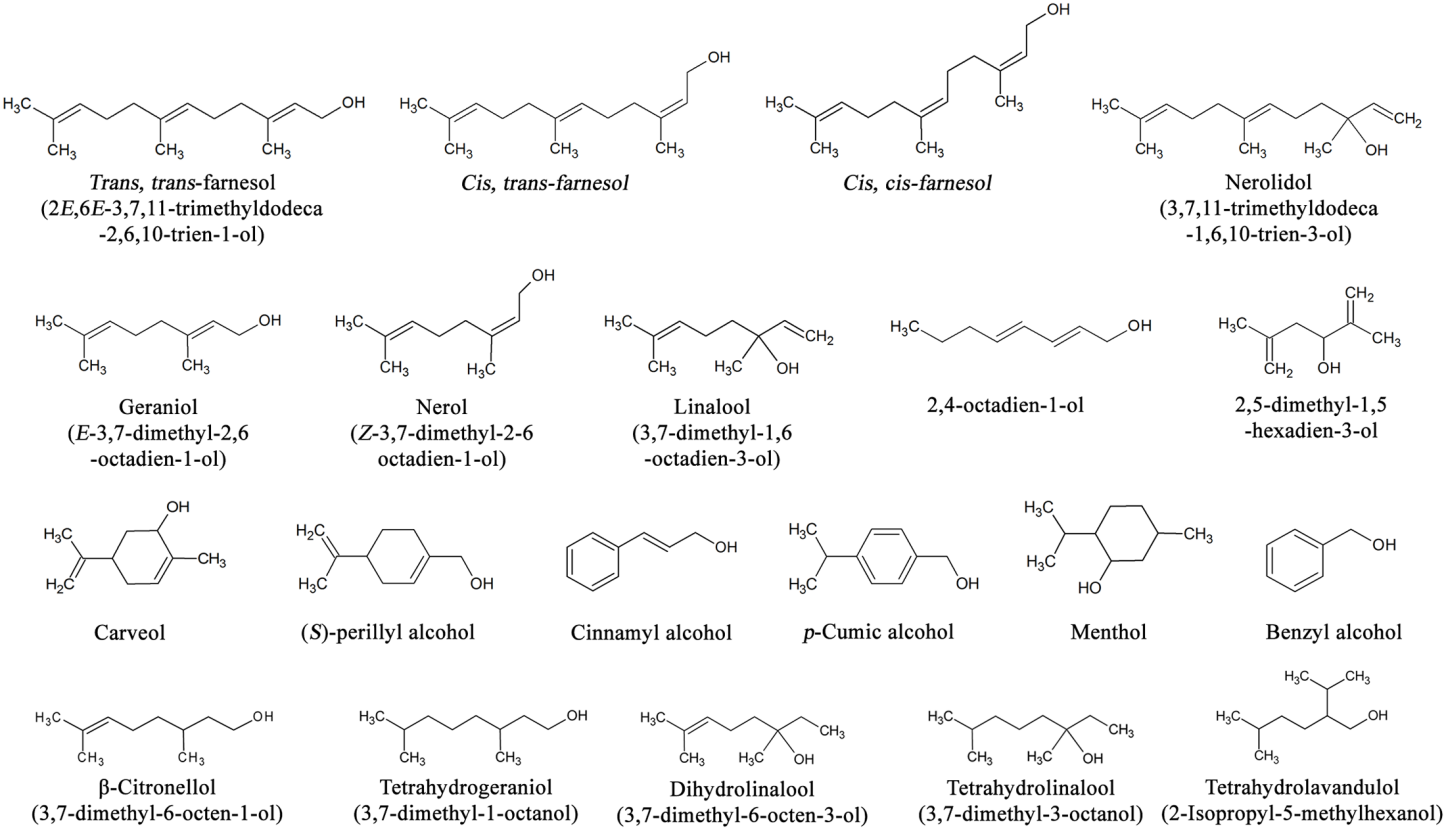
List of substrates tested for substrate specificity of *P*. *minus* farnesol dehydrogenase.

The ability of *P*. *minus* farnesol dehydrogenase (0.2 μg) to oxidize a range of substrates was determined at pH 9.5 (Fig 1). Enzyme activities were evaluated with different alcohol as substrates namely allylic alcohols (*trans*, *trans*-farnesol, *cis*, *trans*-farnesol, *cis*, *cis*-farnesol, nerolidol, geraniol, nerol, linalool, 2,4-octadien-1-ol, and 2,5-dimethyl-1,5-hexadien-3-ol), non-allylic alcohols (β-citronellol, and 3,7-dihydrolinalool), aromatic alcohols (carveol, (*S*)-perillyl alcohol, cinnamyl alcohol, *p*-cumic alcohol, borneol, and menthol), and aliphatic alcohols (tetrahydrogeraniol, tetrahydrolinalool, tetrahydrolavandulol, ethanol, and methanol) at concentrations of 1.0 mM, respectively. The enzyme activity was measured as described before. The effect of different substrates concentration, ranging from 0.5 mM to 1.5 mM with 0.25 increment on enzyme activity was estimated under optimal assay conditions (35°C, pH 9.5 and 5 min). The relative rate of oxidation for each substrate was determined as the percent of the enzyme activity measured with *trans*, *trans*-farnesol which was considered to correspond to 100%. The kinetic parameters (Michaelis-Menten constant (*K*_m_) and maximal reaction velocity (*V*_max_) were determined by linear regression from double-reciprocal plots according to Lineweaver-Burk [46]. The *K*_m_ and *V*_max_ were expressed in mM and μmol·min^−1^, respectively.

## Results and Discussion

### Purification of farnesol dehydrogenase from *P*. *minus* leaves

**Fig 2:**
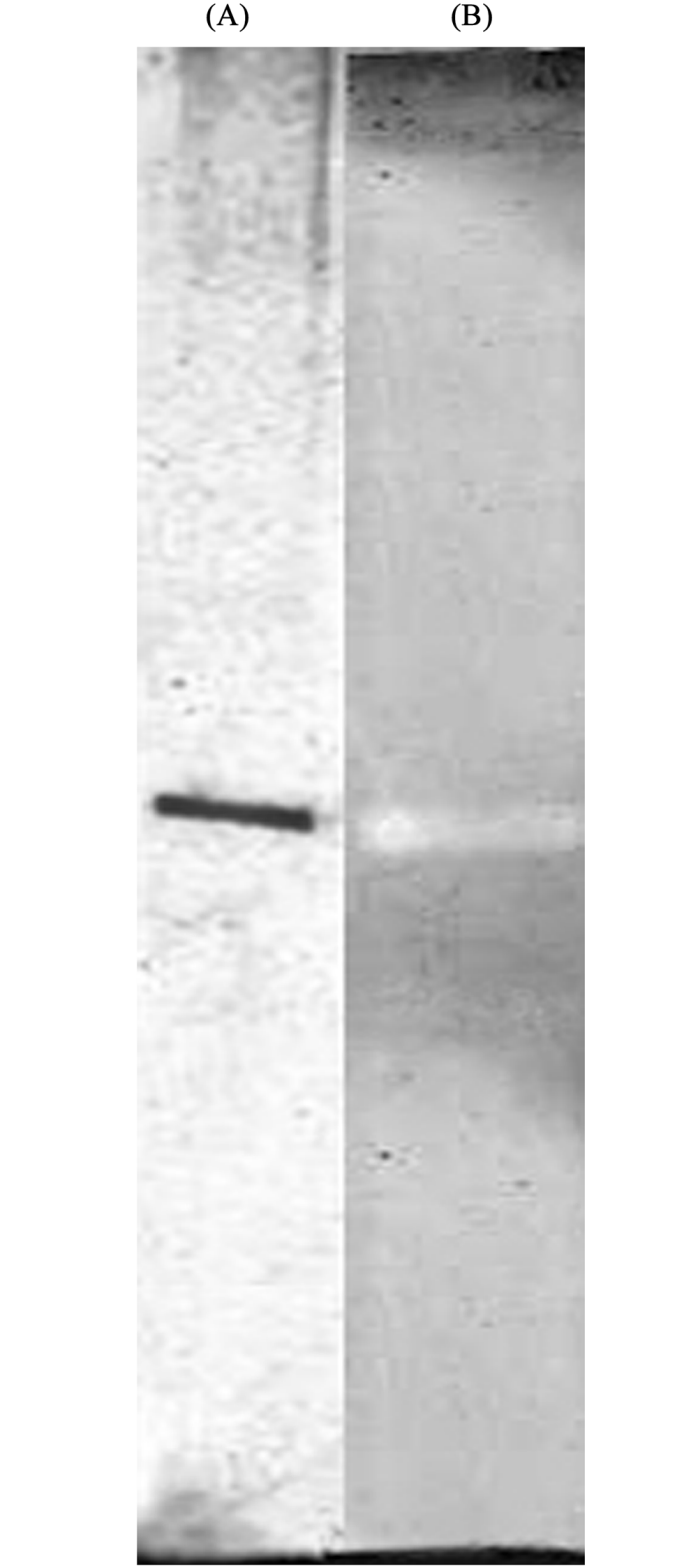
Native-PAGE of the purified farnesol dehydrogenase from *P*. *minus*. Purified enzyme (33 μg) was subjected to electrophoresis in the absence of SDS with 12.5% gel at pH 8.8. Protein gel were stained by silver stain (A) and activity stain (B).

In plants, farnesol dehydrogenase activity has been identified in *A*. *thaliana* [26], chicory [47], sweet potato root tissue [11], and orange flavedo [48]. Recently, a gene on chromosome 4 of the Arabidopsis genome (At4g33360), called *FLDH* was shown to encode a NAD^+^-dependent dehydrogenase that oxidizes farnesol more efficiently than other prenyl alcohol substrates [26]. In this study, NAD(P)^+^-dependent farnesol dehydrogenase from *P*. *minus* leaves has been purified and characterized. *P*. *minus* farnesol dehydrogenase was purified with a high yield (3.2%) by 6 chromatographic steps, including three successive runs on a gel filtration chromatography. All farnesol dehydrogenase activities were recovered in the flow-through fractions of the DEAE-Toyopearl 650M, SP-Toyopearl 650M and Super-Q Toyopearl 650M with increased specific activity. The last step of purification was performed with a TSK-gel GS3000SW gel filtration chromatography. The purification scheme and their results are summarized in Table 1. The purification procedures purified farnesol dehydrogenase about 234-fold with about 3.2% recovery of the enzyme activity. The third run on the TSK-gel GS3000SW resulted in fractions containing farnesol dehydrogenase activity that gave a single protein band by native-PAGE at the same position on the gel where the enzyme activity was detected (Fig 2), strongly suggesting that the farnesol dehydrogenase enzyme from *P*. *minus* was purified to homogeneity. The three successive run on TSK-gel GS3000SW was crucial to maintain the homogeneity of the enzyme where elimination of these step will resulted in impurity of the protein sample.

**Table 1:** Summary of purification of farnesol dehydrogenase from *P*. *minus* leaves.

Purification step	Total activity (U)	Total protein (mg)	Specific activity (U·mg^-1^)	Purification (fold)	Yield (%)
Cell-free extract	7.81	6600	1.2 × 10^−3^	1.0	100.0
DEAE Toyopearl 650M	7.22	946	7.6 × 10^−3^	6.5	92.5
SP Toyopearl 650M	6.44	349	1.7 × 10^−2^	14.0	82.5
Super-Q Toyopearl 650M	2.70	67	4.0 × 10^−2^	34.0	34.6
1^st^ TSK-Gel GW3000SW	0.65	5	1.3 × 10^−1^	107.0	8.4
2^nd^ TSK-Gel GW3000SW	0.48	2	2.4 × 10^−1^	204.0	6.2
3^rd^ TSK-Gel GW3000SW	0.25	0.9	2.7 × 10^−1^	234.0	3.2

### Determination of molecular mass and isoelectric point

**Fig 3:**
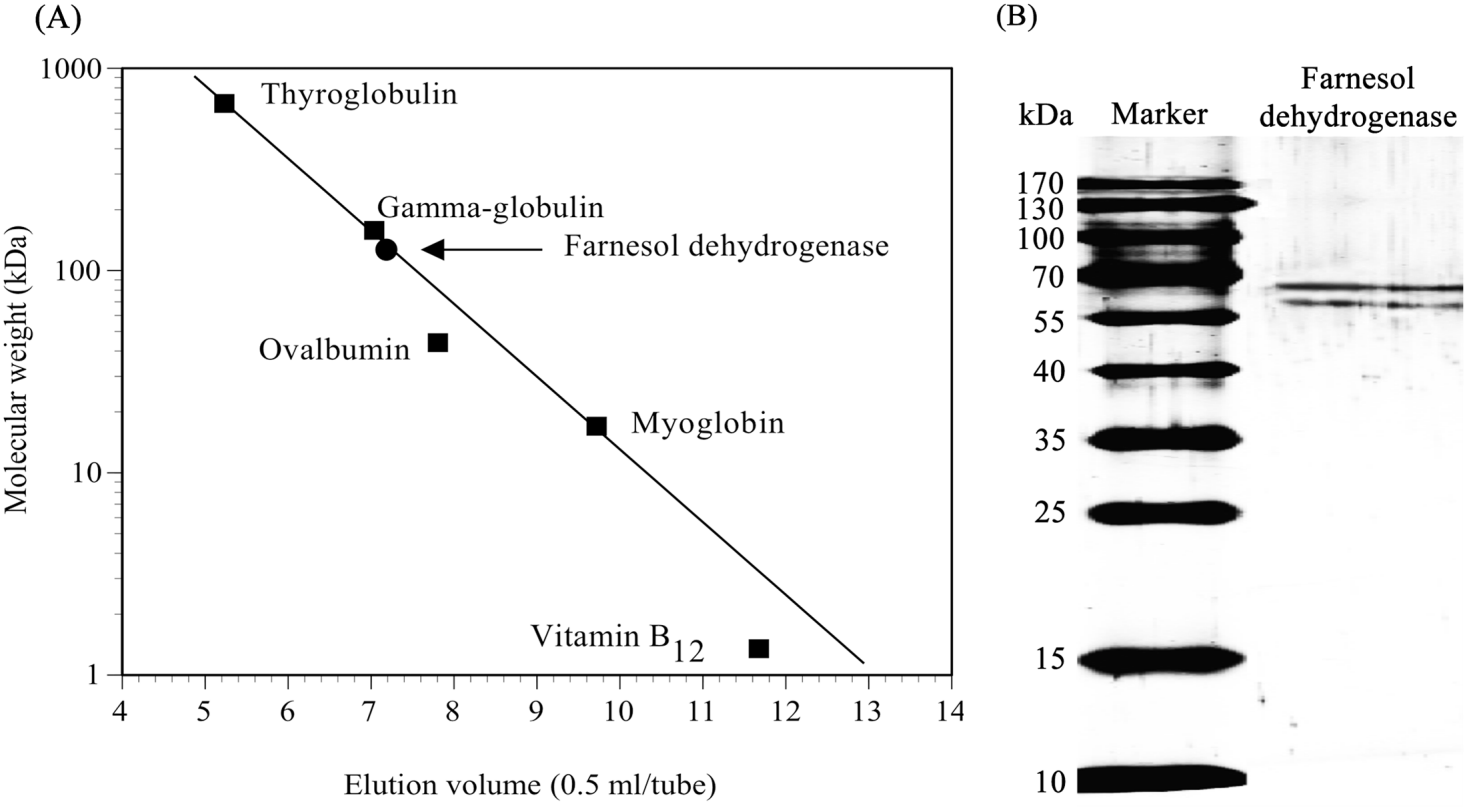
Determination of molecular mass of the farnesol dehydrogenase from *P*. *minus*. (A) Estimation of native molecular mass of farnesol dehydrogenase by TSK-gel GS3000SW column. Experimental conditions are described in “Materials and Methods”. Standard protein marker (■): thyroglobulin (670 kDa), γ-globulin (158 kDa), ovalbumin (44 kDa), myoglobin (17 kDa), and vitamin B_12_ (1350 Da). Farnesol dehydrogenase (●). (B) SDS-PAGE analysis of purified farnesol dehydrogenase. Purified enzyme and standard proteins were subjected to electrophoresis in the presence of SDS with a 12.5% polyacrylamide gel. The PageRuler™ Prestained Protein Ladder, ~10–170 kDa (SM0671) (Fermentas) was used as the molecular marker.

The native molecular mass of the purified enzyme was determined by gel filtration (TSK-gel GS3000SW). The farnesol dehydrogenase showed a relative molecular mass of 130 kDa (Fig 3A). The molecular weight of the monomers of farnesol dehydrogenase were 65 kDa and 70 kDa (Fig 3B), suggesting that *P*. *minus* farnesol dehydrogenase appears to be a heterodimeric enzyme. In contrast, both of farnesol dehydrogenase from *I*. *batatas* and *A*. *aegypti* are homodimers with molecular masses of 60 and 90 kDa, respectively [11, 12]. In addition, several plant alcohol dehydrogenases have been reported to be heterodimeric [39, 49, 50] or homodimeric enzymes [51–53]. *Rauwolfia serpentina* acyclic monoterpene primary alcohol dehydrogenase [54] and the *C*. *lactis* geraniol dehydrogenase [55] are both reported to function as monomers.

The purified farnesol dehydrogenase was subjected to isoelectric focusing and developed by silver staining. One band was visualized, that showed a pI value of 6.8 (S1 Fig). The pI value for *P*. *minus* farnesol dehydrogenase is different from other reported farnesol dehydrogenases. The calculated pI values for *A*. *thaliana* and *A*. *aegypti* farnesol dehydrogenases are 8.27 and 7.06, respectively [11, 12]. The lower pI suggests that farnesol dehydrogenase from *P*. *minus* is rich in acidic amino acid residues.

### Analysis of the protein sequence by MALDI-TOF/TOF-MS

Dialyzed farnesol dehydrogenase was subjected to in-solution digestion with trypsin. Four peptides with m/z values of 2223.04, 2435.06, 3652.21 and 3685.77 were further analyzed with MALDI-TOF/TOF-MS spectrometry and the amino acid sequence of each peptide was determined (Table 2). All 4 amino acid sequences (A,B,C and D) showed similarity (30–70%) to several predicted oxidoreductases and terpene alcohol dehydrogenases, including *P*. *putida p*-cumic alcohol dehydrogenase, *M*. *piperita* (-)-isopiperitenol dehydrogenase, *C*. *defragrans* geraniol dehydrogenase and *S*. *indicum* (+)-neomenthol dehydrogenase. The homology comparison of the peptide sequence (A) and (B) showed a putative conserved domain for Rossmann-fold NAD(P)-binding domain-containing protein. Database searches showed that the peptides of the purified *P*. *minus* farnesol dehydrogenase shared no significant similarity with any reported farnesol dehydrogenases enzymes; however, peptide fragment (A) and (D) showed less than 45% identities to NADP^+^-dependent farnesol dehydrogenase 2 of *Aedes aegypti* and partial farnesol dehydrogenase from *Diploptera punctata*. These results suggested that the purified enzyme might be a novel farnesol dehydrogenase.

**Table 2:** Identification of tryptic peptides from *P*. *minus* farnesol dehydrogenase.

Species	Peptide	E value	Identity (%)
(A) The homology comparison of the peptide sequence from *P*. *minus* farnesol dehydrogenase showed a putative conserved domain for NADB_Rossmann superfamily by a BLASTp search.			
*P*. *minus*	KVWLITGCSTGFGKELTLAALKRGDKVIATARTPAKL	nd.	nd.
*Ricinus communis* ^1^	**KVW**F**ITG**A**S**R**GFG**RIW**T**E**AAL**A**RGDK**VA**ATAR**QL**A**S**L**	2.0×10^-^^12^	68
*Brassica rapa* ^2^	**KV**V**LITG**V**S**K**G**L**G**RA**L**S**L**EMA**KRG**HT**VI**GC**ART**QE**KL**	2.0×10^-^^9^	57
*Musa acuminata* ^3^	**K**TV**LVTG**V**S**R**G**L**G**RA**L**S**L**ELAR**RG**HA**VI**GC**AR**S**P**D**K**V	1.0×10^-^^8^	46
*Nicotiana sylvestris* ^4^	**KV**V**MVTG**A**S**S**G**I**G**R**E**FS**L**DLA**K**S**G**CR**IIA**A**AR**RVDR**L**	2.0×10^-^^8^	43
*Pseudomonas putida* ^5^	**KV**AI**VTG**AA**TG**I**G**NAIVRSY**L**AE**G**A**KVV**	3.0×10^-^^5^	39
*Aedes aegypti* ^6^	**KV**AV**VTG**S**S**S**G**I**G**AAIAKDLA**K**A**G**MV**VV**GL**AR**	2.0×10^-^^4^	38
*Arabidopsis thaliana* ^7^	---**LVTG**STGYL**G**AR**L**CHVL**L**R**RG**HS**V**R**A**LV**R**RTSD**L**	5.0×10^-^^4^	35
*Mentha x piperita* ^8^	**KV**AI**VTG**GAS**G**I**G**EVTARLFAE**RG**ARA**V**VI**A**	5.0×10^-^^3^	29
(B) The homology comparison of the peptide sequence from *P*. *Minus* farnesol dehydrogenase showed a putative conserved domain for NADB_Rossmann superfamily by a BLASTp search.			
*P*. *minus*	RAFLPHMRARRSGVIALIGS	nd.	nd.
*Coccomyxa subellipsoidea* ^9^	Q**A**V**LPHMRA**AQ**SG**Q**I**I**NI**T**S**LVGFSAIP	5.0×10^-^^7^	50
*Solanum lycopersicum* ^10^	---V**PHM**AS**RRSG**S**I**VN**VGS**VVGKVSTP	5.0×10^-^^7^	48
*Oryza sativa Japonica* ^11^	**RA**VA**PHM**AS**RRSG**R**V**VN**VGS**VVGTAATP	1.0×10^-^^6^	46
*Castellaniella defragrans* ^12^	--RIAGVGVCHTDL**V**CRD**G**FP	6.9	32
(C) The homology comparison of the peptide sequence from *P*. *minus* farnesol dehydrogenase showed no putative conserved domain by a BLASTp search.			
*P*. *minus*	KQAELLTRQLSEVHDEAETVIRL	nd.	nd.
*Citrus sinensis* ^13^	**KQ**S**ELL**SKLTR**Q**LSIHD	5.0×10^-^^8^	67
*Sesamun indicum* ^14^	-------EIHE**N**SVA**EAETVIR**	3.0×10^-^^6^	60
(D) The homology comparison of the peptide sequence from *P*. *Minus* farnesol dehydrogenase showed no putative conserved domain by a BLASTp search.			
*P*. *minus*	KTPAAAAIAHHAAVDGKQPGDPVKA	nd.	Nd.
*Rhodococcus opacus* ^15^	--------**A**INE**AV**AAQHR**GD**V**VKA**	7.1×10^-^^2^	47
*Diploptera punctata* ^16^	-----**AAI**TQQLVK**DG**	1.1	45

^1^ (XP_002534918.1) putative 3-oxoacyl-[acyl-carrier-proten] reductase of *Ricinus communis*

^2^ (XP_009144454.1) predicted carbonyl reductase family member 4 of *Brassica rapa*

^3^ (XP_009415001.1) predicted uncharacterized oxidoreductase YMR226C isoform X1 of *Musa acuminata subsp*. *Malaccensis*

^4^ (XP_009792301.1) Predicted dehydrogenase/reductase SDR family member 7-line of *Nicotiana sylvestris*

^5^ (AAB62297.1) *p*-cumic alcohol dehydrogenase of *Pseudomonas putida*

^6^ (ADB03640.1) NADP^+^-dependent farnesol dehydrogenase 2 of *Aedes aegypti*

^7^ (AEE86213.1) Rossmann-fold NAD(P)-binding domain-containing protein of *Arabidopsis thaliana*

^8^ (AAU20370.1) (-)-isopiperitenol dehydrogenase of *Mentha x piperita*

^9^ (XP_005650109.1) NAD(P)-binding protein from *Coccomyxa subellipsoidea* C-169

^10^ (XP_004231947.1) predicted NADPH-dependent -1-acylihydroxyacetone phosphate reductase-like of *Solanum lycopersicum*

^11^ (ABA99950.2) oxidoreductase, short chain dehydrogenase/reductase family protein, expressed from *Oryza sativa Japonica* group

^12^ (CCF55024.1) geraniol dehydrogenase of *Castellaniella defragrans*

^13^ (KDO39154.1) hypothetical protein CISIN_1g030018mg of *Citrus sinensis*

^14^ (XP_011091715.1) predicted (+)-neomenthol dehydrogenase of *Sesamun indicum*

^15^ (AII08455.1) geraniol dehydrogenase *(Rhodococcus opacus)*

^16^ (AHZ20737.1) farnesol dehydrogenase, partial *(Diploptera punctata*)

^a^ nd.–not determined

### Effects of temperature and pH

**Fig 4:**
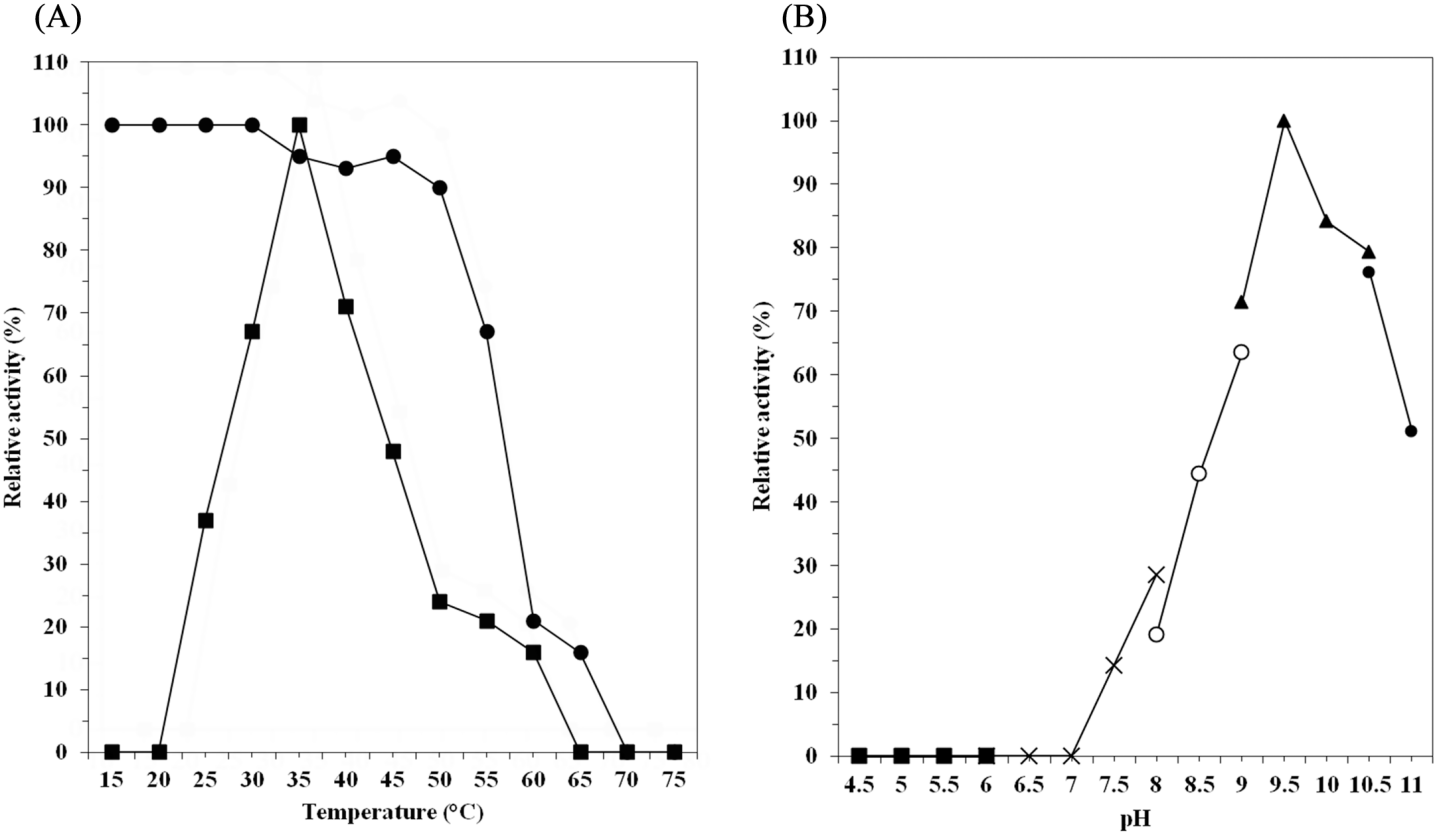
Effects of temperature and pH. (A) Effects of temperature on enzyme activities of farnesol dehydrogenase and stability of the enzyme. The temperature stability was determined by incubating the purified enzymes at a temperature in the range of 25–70°C for 10 min at pH 7.5 (100 mM tricine-NaOH containing 2.5 mM 2-ME). The residual farnesol dehydrogenase activity was assayed as described in “Materials and Method”. The optimal temperature was determined by performing the standard enzyme assay as described in “Materials and Methods,” except that the reaction temperature was varied. Thermo stability (●), optimal temperature (■). (B) Effect of pH on enzyme activity of farnesol dehydrogenase. Enzyme activity was assayed under the standard assay conditions, except that the following buffers were used at a final concentration of 100 mM in the incubation mixture: citrate buffers (■), potassium phosphate buffers (×), Tris-HCl buffers (○), glycine-NaOH buffers (▲), and carbonate buffers (●).

The effects of pH and temperature on the enzyme activity and stability of *P*. *minus* farnesol dehydrogenase was investigated. The optimal temperature of the enzyme was found to be 35°C (Fig 4A), which is comparable to the optimal temperature of farnesol dehydrogenases from *I*. *batatas* [11] and *A*. *aegypti* [12] (25°C-30°C). The residual activity of farnesol dehydrogenase was measured after heat treatment at various temperatures for 10 min in 2.5 mM 2-ME containing tricine-NaOH buffer (100 mM, pH 7.5) (Fig 4A). The enzyme showed the highest activity at 35°C, and remained stable at temperatures up to 50°C. The enzyme activity of *P*. *minus* farnesol dehydrogenase declined rapidly at temperatures greater than 50°C. Rapid inactivation of enzyme activity was observed when the enzyme activity was measured from 55 to 65°C. Approximately 70% of the activity at was observed at 55°C and only 16% of the activity left remained at 65°C. The enzyme activity was completely lost at 70°C.

The optimal pH for *P*. *minus* farnesol dehydrogenase was found to be 9.5 (Fig 4B). At pH below 8.5 or above 10.0, the enzyme activity declined sharply, suggesting that *P*. *minus* farnesol dehydrogenase has a narrow alkaline pH optimum. This result is comparable to the optimal pH for farnesol dehydrogenases from *I*. *batatas* [11] and *A*. *aegypti* [12] (pH 9.5–11), and to the terpene alcohol dehydrogenases [39, 49, 54, 56, 57] (pH 9.0–9.5).

### Effects of metal ions and inhibitors

Oxidation of *trans*, *trans*-farnesol by *P*. *minus* farnesol dehydrogenase was inhibited completely by heavy metal ions and sulfhydryl agents such as *p*-chloromercuribenzoate, 5,5’-dithiobis (2-nitrobenzoic acid), and iodoacetoamide (Table 3). The activity was also completely inhibited by EDTA and 1,10-phenanthroline, a major chelating agent of Zn ion. Chelating agents of the Fe ion, 2, 2’-dipyridyl and sodium azide caused approximately 25–50% inhibition in farnesol dehydrogenase activity. Terpene alcohol dehydrogenases were also inhibited by sulfhydryl group inhibitors and heavy metals [11, 39, 49, 54, 56, 58–60]. Inactivation of *P*. *minus* farnesol dehydrogenase by sulfhydryl reagents suggested that the sulfhydryl group of the enzyme is essential for catalytic activity. The inhibition of activity by EDTA and other metal chelators also suggests the participation of one or more metal ions in the enzyme activity.

**Table 3:** Effects of inhibitors on the farnesol dehydrogenase activity. The enzyme was preincubated for 5 min at 35°C with the various reagents before addition of the substrate. Each reagent was added at the final concentration as indicated.

Reagent (1.0 mM)	Relative activity (%)
None	100
2,2-Dipyridil	48
Iodoacetamide	34
Sodium azide	75
5,5’-Dithiobis (2-nitrobenzoic acid)	0
*p*-Chloromercuribenzoate	0
EDTA	0
1, 10-Phenanthroline	0
Lithium chloride	0
Silver nitrate	0
Zinc chloride	0
Magnesium chloride	27
Calcium chloride	76
Iron (III) chloride	0
Cuprum sulphate	0

### Substrate specificity and Michaelis-Menten constants

*P*. *minus* farnesol dehydrogenase utilized both NAD^+^ and NADP^+^ as coenzymes. The utilization of NAD^+^ by the enzyme was 5 times more efficient than for NADP^+^, while the *K*_m_ value for NADP^+^ was over 54-fold greater than the corresponding value for NAD^+^ (Table 4). Both NAD^+^ and NADP^+^ also can be utilized as coenzymes for several terpene alcohol dehydrogenases, such as *M*. *piperita* isopiperitenol dehydrogenase [59], *N*. *tabacum* allylic alcohol dehydrogenase [61], and *S*. *officinalis* borneol dehydrogenase [60]. On the other hand, farnesol dehydrogenases from *I*. *batatas* [11] and *A*. *aegypti* [12] and several plant terpene alcohol dehydrogenases [39, 56, 57, 58, 62]; acyclic monoterpene primary alcohol DH [49, 54] were highly specific for NADP^+^. Other terpene alcohol dehydrogenases such as *C*. *lactis* geraniol dehydrogenase[55], Pseudomonas perillyl alcohol dehydrogenase [63], *Nepeta racemosa* nepetalactol oxidoreductase[49], and *L*. *x intermedia* borneol dehydrogenase [64] were very specific to NAD^+^.

**Table 4:** Substrate specificity, coenzyme specificity, and kinetic parameters of *P*. *minus* farnesol dehydrogenase.

Substrate	Relative activity (%)	*K*_m_ value (mM)	*V*_max_ value (μmol/ml)
*Trans-trans*-farnesol	100	0.17	0.24
*Cis-trans*-farnesol	66	0.33	0.26
*Cis-cis*-farnesol	47	0.42	0.25
Nerodilol	36	1.00	0.29
Geraniol	37	12.50	0.63
Nerol	0	n.d	n.d
Carveol	32	0.71	0.45
(*S*)-Perillyl alcohol	32	0.77	0.20
Cinnamyl alcohol	35	1.04	0.15
*p*-Cumic alcohol	0	n.d	n.d
NAD^+^	100	0.74	0.38
NADP^+^	22	40.00	2.50

n.d- not determined.

The *P*. *minus* enzyme showed the highest activity toward *trans*, *trans*-farnesol, *cis*, *trans*-farnesol and *cis*, *cis*-farnesol. Nerolidol, geraniol, (*S*)-perillyl alcohol, cinnamyl alcohol, and carveol were also oxidized at rates less than 40% compared to *trans*, *trans*-farnesol (Table 4). On the other hand, *P*. *minus* farnesol dehydrogenase did not react with other terpene alcohols such as *p*-cumic alcohol, borneol, linalool, or menthol. No detectable activity was observed with β-citronellol, 3,7-dihydrolinalool, benzyl alcohol, 2,4-octadien-1-ol, or 2,5-dimethyl-1.5-hexadien-3-ol. Tetrahydrogeraniol, tetrahydrolinalool, tetrahydrolavandulol, ethanol, and methanol were inert as substrates. The kinetic parameters determined for farnesol dehydrogenase were shown in Table 4. No reduction reaction was observed.

*P*. *minus* farnesol dehydrogenase oxidizes not only *trans*, *trans*-farnesol, but also its geometrical and structural isomers. The geometrical isomers of *trans*, *trans*-farnesol, (*cis*, *trans*-farnesol and *cis*, *cis*-farnesol) were oxidized at 66% and 47% of the rate observed for *trans*, *trans*-farnesol. The *K*_m_ values of *cis*, *trans*-farnesol and *cis*, *cis*-farnesol were approximately 2-fold and 2.5-fold higher than for *trans*, *trans*-farnesol, respectively. Nerolidol, a structural isomer of *trans*, *trans*-farnesol, was also oxidized at a lower rate compared to *trans*, *trans*-farnesol. The *K*_m_ value for nerolidol was approximately 6-fold higher than *trans*, *trans*-farnesol. In addition, farnesol dehydrogenases from *A*. *aegypti* and *I*. *batatas* oxidize *cis*, *cis*-farnesol [12] and *cis*, *trans*-farnesol [11], respectively, at lower rates compared to *trans*, *trans*-farnesol. These results indicate that *P*. *minus* farnesol dehydrogenase is capable of distinctly recognizing the geometrical and structural isomers of substrates, similar with other farnesol dehydrogenases [11, 12] and geraniol dehydrogenases [39, 55, 58, 62].

The positions of the double bonds at carbon positions 2 and 6 in the substrate also play a significant role in the substrate selectivity of *P*. *minus* farnesol dehydrogenase. Besides *trans*, *trans*-farnesol and its isomers, *P*. *minus* farnesol dehydrogenase selectively oxidizes geraniol ((*trans*)-3,7-dimethyl-2,6-octadien-1-ol) but at a much lower rate. Moreover, linalool, a structural analog of geraniol, that has double bond in position 1 and 6 was not oxidized. Neither non-allylic nor aliphatic analogs of geraniol, β-citronellol (3, 7-dimethyl-6-octen-1-ol) and tetrahydrogeraniol (3, 7-dimethyl-1-octanol), were substrates for *P*. *minus* farnesol dehydrogenase. These results were consistent with previous reports of farnesol dehydrogenases from *A*. *aegypti* [12] and *I*. *batatas* [11]. The *K*_m_ values for geraniol and β-citronellol of *A*. *aegypti* farnesol dehydrogenase [12] was about 2.3-fold and 1.3-fold higher than obtained with *trans*, *trans*-farnesol as substrate, respectively. The relative activities of geraniol and β-citronellol for *I*. *batatas* farnesol dehydrogenase were 57% and 37% of the rate observed with *trans*, *trans*-farnesol, respectively [11].

Interestingly, *P*. *minus* farnesol dehydrogenase also oxidized several aromatic alcohols such as carveol, (*S*)-perillyl alcohol, and cinnamyl alcohol. On the other hand, aromatic alcohols, such as benzyl alcohol and *p*-cumic alcohol (which does not have an alkenyl group near the carbinol) were not oxidized by *P*. *minus* farnesol dehydrogenase.

*P*. *minus* [39], *O*. *basilicum* [57], and *C*. *defragrans* [65] geraniol dehydrogenases, as well as *P*. *putida* benzyl alcohol dehydrogenase [66] and *P*. *putida* MB-1 allylic alcohol dehydrogenase [67] show broad substrate specificity with substrates containing an allylic double bond or with an aromatic ring attached to the carbinol carbon. Benzyl alcohol dehydrogenase from *Acinetobacter calcoaceticus* oxidized not only a range of aromatic alcohols related to benzyl alcohol but also the allylic alcohol moieties in perillyl, cinnamyl, and coniferyl alcohols [68]. However, 2-phenylethanol, which does not have an alkenyl group near the carbinol, was not a substrate for this enzyme. MacKintosh and Fewson suggested that for cinnamyl and coniferyl alcohols, the alkenyl group, which is located between the aromatic ring and the carbinol center may help correctly position the alcohol in the active site [68].

## Conclusion

In this study, we purified the farnesol dehydrogenase enzyme from *P*. *minus* leaves and characterized its biochemical properties. This enzyme has several different biochemical characteristics from the previously identified farnesol dehydrogenases regarding the substrate profile, isoelectric point, molecular weight and utilization of both NAD^+^ and NADP^+^ as coenzymes. Furthermore, a MALDI-TOF/TOF-MS analysis of the purified enzyme revealed no homology to any known farnesol dehydrogenase. Altogether, these data suggest that the purified enzyme is a novel form of NAD(P)^+^-dependent farnesol dehydrogenase. Studies of the enzyme’s substrate specificity and kinetic parameter results suggest that the selectivity of substrate specificity of *P*. *minus* farnesol dehydrogenase depends on the *cis* or *trans* configuration, the number of the isoprene units, and the position of double bonds of the substrate. In addition, *P*. *minus* farnesol dehydrogenase exhibited no activity against aliphatic alcohols demonstrating that this enzyme was specific for substrates containing an allylic double bond. The complete sequence, structure and functional analysis of *P*. *minus* farnesol dehydrogenase may lead to the discovery of novel strategies for development of integrated pest management in plant by engineering the transgenic plants using insect-resistant genes to fight insect pests [69]. Nevertheless, further studies on the hormonally linked relationship between plants and insect pest could well indicate the method for utilizing the chemicoecological interaction in integrated pest management [28].

## Supporting Information

S1 Fig Isoelectric focusing of purified farnesol dehydrogenase. The arrows indicate the protein bands approximately at pI 6.8. ReadyStrip IPG strips are preprinted to indicate anode end and pH range. A barcode is printed toward the pointed end of the strip holder.
(TIF)
