# Supplementary figures and images for: Purification and Characterization of a Novel NAD(P)+-Farnesol Dehydrogenase from Polygonum minus Leaves
Source: PLoS One. 2015 Nov 23;10(11):e0143310. doi: 10.1371/journal.pone.0143310 (PMC4657912; doi:10.1371/journal.pone.0143310)

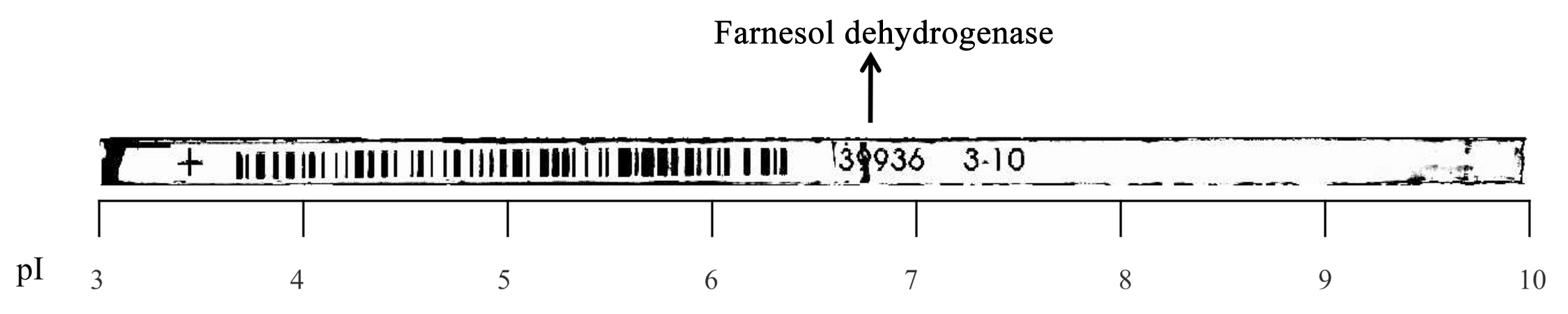

Supplement: S1 Fig — The arrows indicate the protein bands approximately at pI 6.8. ReadyStrip IPG strips are preprinted to indicate anode end and pH range. A barcode is printed toward the pointed end of the strip holder. (TIF) [file pone.0143310.s001.tif]
